# Factors influencing survival after kidney transplant failure

**DOI:** 10.1186/2047-1440-3-18

**Published:** 2014-09-24

**Authors:** Jennifer A McCaughan, Christopher C Patterson, Alexander P Maxwell, Aisling E Courtney

**Affiliations:** 1 Regional Nephrology Unit, Belfast City Hospital, Belfast BT9 7AB, UK; 2 Nephrology Research Group, Queen’s University, Belfast BT9 7AB, UK; 3 Center for Public Health, Queen’s University, Belfast BT12 6BA, UK

**Keywords:** Kidney transplantation, Survival, Graft failure, Re-transplantation

## Abstract

**Background:**

The failure of a kidney transplant is now a common reason for initiation of dialysis therapy. Kidney transplant recipients commencing dialysis have greater morbidity and mortality than transplant-naïve, incident dialysis patients. This study aimed to identify variables associated with survival after graft failure.

**Methods:**

All recipients of first, deceased donor kidney transplants performed in Northern Ireland between 1986 and 2005 who had a functioning graft at 12 months were included (*n* = 585). Clinical and blood-derived variables (age, gender, primary renal disease, diabetic status, smoking status, human leukocyte antigen (HLA) mismatch, acute rejection episodes, immunosuppression, cardiovascular disease, graft survival, haemoglobin, albumin, phosphate, C reactive protein, estimated glomerular filtration rate (eGFR), rate of eGFR decline, dialysis modality, and access) were collected prospectively and investigated for association with re-transplantation and survival. The association between re-transplantation and survival was explored by modelling re-transplantation as a time-dependent covariate.

**Results:**

Median follow-up time was 12.1 years. Recipients with a failing graft (158/585) demonstrated rapid loss of eGFR prior to graft failure, reducing the time available to plan for alternative renal replacement therapy. Median survival after graft failure was 3.0 years. In multivariate analysis, age and re-transplantation were associated with survival after graft failure. Re-transplantation was associated with an 88% reduction in mortality.

**Conclusions:**

Optimal management of kidney transplant recipients with failing grafts requires early recognition of declining function and proactive preparation for re-transplantation given the substantial survival benefit this confers. The survival benefit associated with re-transplantation persists after prolonged exposure to immunosuppressive therapy.

## Background

The proportion of the incident dialysis population with a failed kidney transplant is increasing each year [1,2]. Despite advances in kidney transplantation which have substantially reduced the rate of early graft loss, the impact on long-term graft survival has been disappointing [3,4]. The morbidity and mortality of kidney transplant recipients commencing dialysis are high [5-11].

Transplant recipients receive intensive supervision from nephrologists following transplantation and review continues throughout the lifespan of the transplant, albeit at a reduced frequency. It is reasonable to anticipate that these individuals should receive optimal care with regard to management of the complications of progressive chronic kidney disease (CKD) and planning for dialysis. The available evidence suggests that this is not the case. Markers of anaemia, nutrition, inflammation, and mineral bone disease are reportedly worse in the failed renal transplant population than in transplant-naïve patients at the initiation of dialysis [7,12-14]. This is despite the fact that one quarter of the incident dialysis population are not known to a nephrologist more than 1 month prior to commencing dialysis therapy [15].

Kidney transplant failure has ramifications for both transplant recipients and healthcare providers. While there is evidence that there may be sub-optimal management of transplant recipients in the pre-dialysis period, limited data has been published to highlight which modifiable clinical parameters are associated with survival following graft failure. We aimed to address this issue in our study.

## Patients and methods

### Patient cohort

All recipients of first, deceased donor transplants undertaken between 1986 and 2005 in Northern Ireland who had self-supporting graft function at 12 months were included. Recipients with graft failure within the first year were excluded as they defaulted to their pre-transplant dialysis modality without a prodromal period during which CKD complications could be managed and preparations made for alternative renal replacement therapy (RRT). Individuals whose grafts failed during follow-up at another centre or who returned to dialysis following urgent graft nephrectomy were also excluded. Recipients were followed up until death or August 1, 2012.

### Transplant data

Clinical data for all recipients and donors is prospectively collected; recorded variables include age, gender, primary renal disease, diabetic status, smoking status, human leukocyte antigen (HLA) mismatch, immunosuppression, acute rejection episodes, cardiovascular disease, graft survival, mode of RRT at graft failure, recipient survival, and cause of death. Primary renal disease is grouped into six categories: glomerulonephritis, chronic pyelonephritis/tubulointerstitial disease, autosomal dominant polycystic kidney disease, diabetic nephropathy, other specified etiologies, and CKD not defined. The diagnosis of cardiovascular disease necessitates physician documentation and objective evidence; for example, a diagnosis of "myocardial infarction" would require clinical documentation with a corresponding electrocardiogram, elevation in cardiac enzymes, or record of confirmatory angiography.

### Graft failure data

Graft failure was defined as the commencement of an alternative mode of RRT. Clinical and blood-derived parameters from the date of graft failure were obtained from a combination of patient records, the laboratory results system, and the regional renal database (a prospective clinical database maintained by nephrologists).

Clinical information was collected on dialysis modality and type of access for dialysis: temporary catheter, tunnelled catheter, polytetrafluoroethylene (PTFE) graft, arteriovenous fistula (AVF), or peritoneal dialysis catheter.

Estimated glomerular filtration rate (eGFR) at the initiation of dialysis therapy was calculated using the four-variable MDRD equation [16]. The rate of decline in eGFR prior to graft failure was calculated by least squares linear regression using a minimum of six values obtained at four monthly intervals.

Serum haemoglobin, phosphate, albumin, and C reactive protein concentrations were recorded within 1 month of graft failure.

### Statistical analysis

In the analysis of graft survival, the factors included in Cox regression analysis were recipient age, primary renal disease, donor age, era of transplantation, HLA mismatch, biopsy-proven acute rejection within 6 months, and type of maintenance immunosuppression.

In the analysis of patient survival, re-transplantation was included as a time-dependent covariate in a Cox’s proportional hazards model. In this model, patients contribute follow-up to the "no re-transplantation" group prior to re-transplantation and then switch to the "re-transplantation" group once a second transplant has occurred. If there is subsequent failure of the re-transplant, follow up continues in the latter group. This model reduces the statistical bias which occurs in a standard Cox’s proportional hazard model. For pre-emptive re-transplants, the time to re-transplantation was recorded as 0. Factors included in multivariate analysis were recipient age, gender, primary renal disease, diabetes mellitus, cardiovascular disease, smoking status, anaemia, hyperphosphatemia, and mode of RRT.

Time to re-transplantation was also analysed using the Cox’s proportional hazards model with death considered as a competing risk. This method is used when follow-up for the outcome of interest (re-transplantation) is no longer possible because of the occurrence of a competing event (death) [17].

SPSS version 20 (SPSS Inc., Chicago, IL) was used for univariate analyses. Stata release 11 (StataCorp, College Station, TX) was used for time-dependent covariate and competing-risks survival time analyses.

### Ethics statement

Ethical approval for this study was granted by the Regional Ethics Committee (12/NI/0178).

## Results

### Patient demographics

There were 707 first, deceased donor kidney transplants performed between 1986 and 2005. Twelve months after transplantation, 585 recipients had functioning grafts; the demographics of this population are reported in Table 1. Prednisolone and azathioprine was the routine dual immunosuppressive therapy in this centre until 1989 when calcineurin inhibitors (CNI) were introduced; CNI-free maintenance regimens were employed in 25% of the recipients. No induction therapy was used during the study period. The median follow-up time was 12.1 years (range 1–26 years).

**Table 1:** Demographics of 585 recipients in the study population

**Variable**	***n***
Recipient variables	
Age, mean (SD), years	41 (16.5)
Male gender	372 (64%)
Primary renal disease	
Glomerulonephritis	121 (21%)
Chronic pyelonephritis/tubulointerstitial disease	119 (20%)
Autosomal dominant polycystic kidney disease	88 (15%)
Chronic kidney disease—not specified	77 (13%)
Diabetic nephropathy	53 (9%)
Other specified etiologies	127 (22%)
Donor variables	
Age, mean (SD), years	36 (16.5)
Male gender	343 (59%)
Transplant variables	
Decade of transplantation	
1986–1995	303 (52%)
1996–2005	282 (48%)
HLA mismatch, mean (SD), no. of antigens	2.2 (1.1)
Ischaemic time, mean (SD), minutes	1,426 (442)
Acute rejection^a^	108 (19%)
CNI-based immunosuppression	461 (79%)

^a^Biopsy-proven acute rejection within 6 months.

### Graft failure

**Figure 1:**
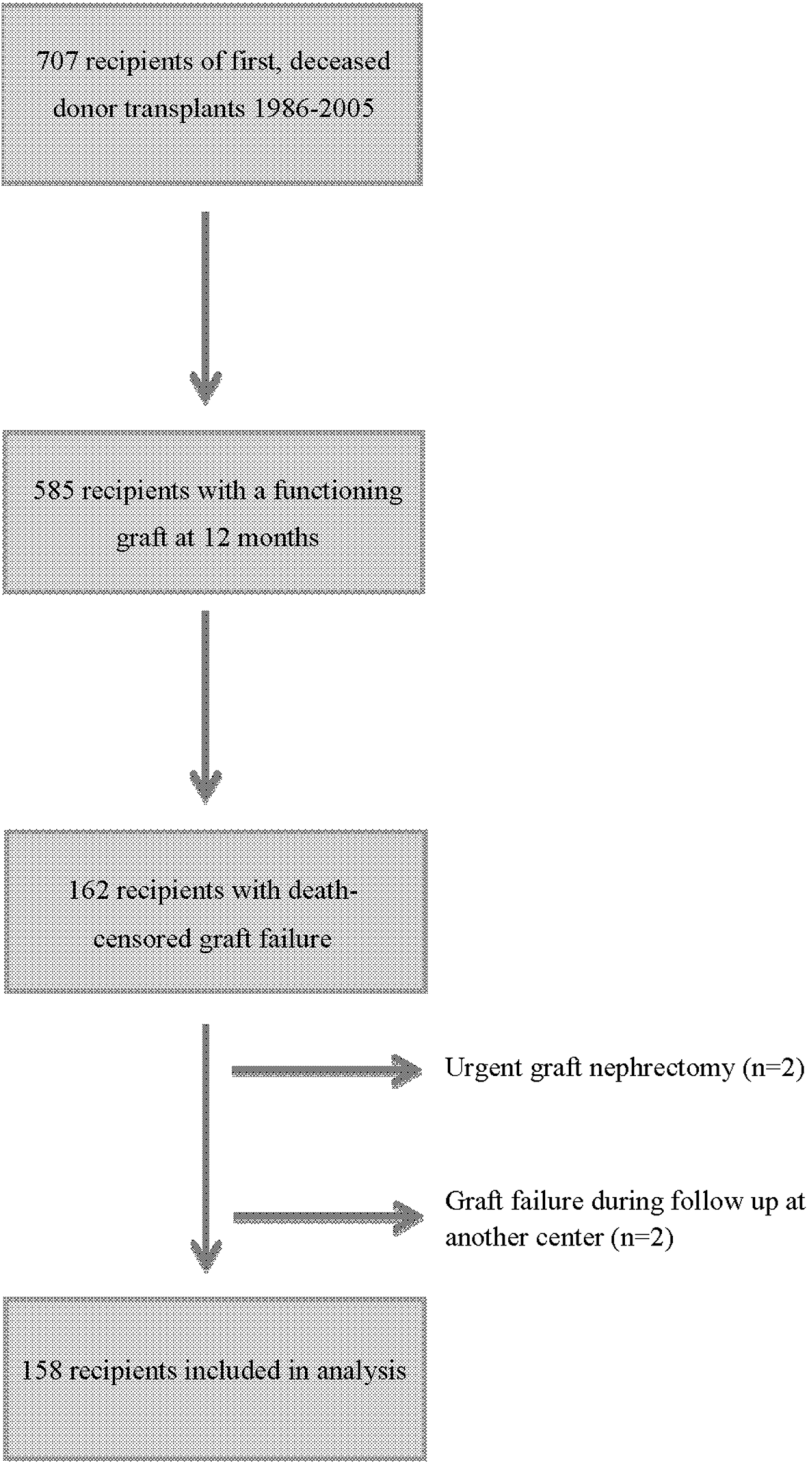
Identification of the study cohort.

There were 162 cases of death-censored graft failure; the median graft survival was 10.4 years (range 1–26 years). Transplant biopsies were performed on 99 recipients with subsequent death-censored graft failure; 10% had late acute rejection, 3% had chronic immunological injury, 23% had interstitial fibrosis/tubular atrophy attributed to CNI toxicity, 22% had recurrence of their primary renal disease, 31% had another histological diagnosis, and 11% were non-diagnostic. Two individuals with graft failure which occurred during follow-up in another centre were not included in further analyses. A further two individuals were excluded because they required urgent graft nephrectomy, one for renal cell carcinoma and the other for refractory malignant hypertension, while maintaining self-supporting graft function (Figure 1). The median follow-up time after graft failure was 4.8 years (range 0–21.8 years).

In multivariate analysis, acute rejection (HR 2.2, *p* < 0.001), donor age (HR 1.2 (per decade), *p* = 0.001), and calcineurin inhibitor-based immunosuppression (HR 2.2, *p* = 0.003) were strongly associated with graft failure.

### Renal replacement therapy

#### Estimated GFR

The eGFR at commencement of alternative RRT was recorded for 143/158 (91%) individuals. The mean eGFR was 9.8 mL/min/1.73 m^2^ (SD 3.9 mL/min/1.73 m^2^). The mean annual loss of eGFR prior to graft failure was 7.9 mL/min/1.73 m^2^ (95% CI 7.0–8.8 mL/min/1.73 m^2^).

#### Dialysis: modality and access

Following graft failure, 154 recipients returned to dialysis therapy and four had pre-emptive transplants. The majority of patients (70%) commenced haemodialysis (Table 2); dialysis modality was unrecorded for nine recipients who died prior to 1997. The haemodialysis cohort was older than the peritoneal dialysis group (45.6 vs 35.8 years, *p* = 0.002), but the prevalence of diabetes mellitus (7% vs 3%, *p* = 0.47) and cardiovascular disease (44% vs 33%, *p* = 0.32) was comparable between the modalities. Almost half of all recipients who started haemodialysis did so with a temporary catheter. All 31 (34%) recipients with a functioning AVF had had this formed prior to transplantation. The annual loss of eGFR prior to graft failure did not differ between recipients who recommenced dialysis via a temporary catheter and those with alternative access (*p* = 0.61).

**Table 2:** Renal replacement therapy and access modality at graft failure

**Mode of RRT**	**Access type**	***n***	**Percentage**
Haemodialysis		111	70
	Temporary catheter	48	
	Arteriovenous fistula	31	
	Tunnelled catheter	28	
	PTFE graft	1	
	Unknown	3	
Peritoneal dialysis	Peritoneal dialysis catheter	34	22
Transplant	Not applicable	4	3
Unknown		9	6

#### Haematological and biochemical parameters

Serum haemoglobin, phosphate and albumin concentrations, and the percentage of individuals attaining K/DOQI targets for CKD5 are shown in Table 3. There were just over a quarter of recipients with haemoglobin, and a fifth with a serum phosphate concentration, in the desired range.

**Table 3:** Haematological and biochemical parameters at graft failure

**Variable**	***n***^**a**^	**Mean**	**SD**	**K/DOQI target**	**Attained target**
Haemoglobin (g/L)	141 (89%)	93	20	100–115	27%
Phosphate (mmol/L)	131 (83%)	1.93	0.59	1.13–1.78	21%
Albumin (g/L)	128 (81%)	33.3	6.5	-	-

^a^Number of participants for whom values were available and percentage of total cohort.

#### Immunosuppression

Immunosuppression was reduced within 12 months following graft failure in 89% of the recipients; 18% were completely weaned from immunosuppressive drugs. Reduction in immunosuppression was not associated with re-transplantation (*p* = 0.7). Graft nephrectomy is not routinely practised but was undertaken in two recipients in the context of refractory acute rejection; one individual had immunosuppression stopped immediately following nephrectomy, while the other was maintained on low dose prednisolone. These individuals are highly sensitised and both have wait-listing times for re-transplantation exceeding 84 months.

### Re-transplantation

There were 56 (35%) patients re-transplanted within the follow-up period; 16 were living donor transplants. The median time to re-transplantation was 27 months (range 0–164 months).

In a univariate competing risks model, older age (HR 0.7 (per decade), *p* <0.001), cardiovascular disease (HR 0.5, *p* = 0.01), diabetes mellitus (HR 0.4, *p* = 0.03), and commencing dialysis with a temporary catheter (HR 0.5, *p* = 0.03) were associated with reduced likelihood of re-transplantation. There was no significant association between HLA mismatch at first transplant (HR 0.9, *p* = 0.11), eGFR at graft failure (HR 0.9, *p* = 0.20), rate of eGFR decline (HR 1.0, *p* = 0.32), commencing peritoneal dialysis (HR 1.5, *p* = 0.20), serum haemoglobin (HR 1.0, *p* = 0.98), serum phosphate (HR 0.8, *p* = 0.46), or serum albumin (HR 1.0, *p* = 0.70). In a Cox regression model, only age (HR 0.71, CI 0.55–0.91) remained associated with re-transplantation; the median age at graft failure in recipients who were re-transplanted was 35 years compared to 52 years in those who did not receive a second graft.

### Recipient survival

There were 65 (41%) deaths within the follow-up period; the median survival time for these recipients after graft failure was 3.0 years and the median age at death was 62 years. The cause of death was unknown for four individuals; 46% (*n* = 28) of the remaining deaths were attributable to cardiovascular disease, 18% (*n* = 11) to infection, 15% (*n* = 9) to malignancy, and the remaining 21% to miscellaneous causes including renal failure (*n* = 5), sudden cardiac arrest (*n* = 3), venous thromboembolism (*n* = 3), respiratory failure secondary to chronic pulmonary disease (*n* = 1), and haemorrhage (*n* = 1). For comparison, the causes of death for recipients with functioning grafts were analysed: 29% were attributable to cardiovascular disease, 22% to malignancy, and 21% to infection. In univariate analysis, diabetes mellitus, cardiovascular disease, higher eGFR, and commencing haemodialysis were associated with mortality; these associations were lost after adjustment for confounders. Ascertainment of targets for serum haemoglobin and phosphate at graft failure was not associated with survival. Multivariate analysis demonstrated that recipient age and re-transplantation were strongly associated with survival (Table 4).

**Table 4:** Cox regression analysis for recipient survival after graft failure

**Variable**	**Hazard ratio**	**95% CI**	***p *** **value**
Recipient age^a^	1.5	1.3–1.9	<0.001
Re-transplantation^b^	0.16	0.06–0.48	0.001
Cardiovascular disease	1.4	0.8–2.6	0.3
Diabetes mellitus	1.3	0.6–2.5	0.5

^a^Per decade.

^b^Modelled as a time-dependent covariate.

## Discussion

Kidney transplantation is transformational for the recipient and cost effective for the healthcare provider [18,19]. There has been notable progress over the past two decades with 1-year death-censored graft survival improving from 85% to 96% [3]. Despite this, a solitary kidney transplant is unlikely to meet the lifelong needs of many recipients, and the number of individuals awaiting re-transplantation has doubled since 1990. This group currently comprise over 15% of the transplant waiting list [20,21].

Kidney transplant recipients returning to dialysis have significantly reduced survival compared to both the transplant-naïve dialysis population and those with functioning renal grafts [5-11]. This is predictable given that this population has acquired the detrimental effects of long-term immunosuppression and also the increased risks associated with a second period of progressive CKD. Cardiovascular disease accounted for 46% of deaths in this cohort. In a study of recipients with failed transplants using USRDS registry data, cardiovascular disease was also the leading cause of death [22]. A greater proportion of deaths in recipients after graft failure were attributable to cardiovascular disease compared to individuals with functioning transplants. This may reflect the prolonged period of CKD, with associated classical and non-classical cardiovascular risk factors, combined with decreased exposure to immunosuppression. The practice in our centre is to minimise immunosuppression following graft failure which may attenuate the development of life-threatening infection and malignancy in this population and explain the reduced proportion of infection and cancer-related deaths.

Re-transplantation was the only clearly modifiable factor in this analysis which improved the survival of recipients with kidney transplant failure; after adjustment for multiple covariates, re-transplantation was associated with an 88% reduction in mortality. While this appears intuitive, analysis of the impact of re-transplantation on survival is complex due to the potential confounding from variables which simultaneously influence the likelihood of re-transplantation and survival. It is also essential to model re-transplantation as a time-dependent covariate in this analysis because of the influence of survival itself on the likelihood of re-transplantation. To the best of our knowledge, this is the first report of the impact of re-transplantation in a cohort with kidney transplant failure who had long-term graft survival and follow-up. Ojo et al. reported a significant survival benefit from re-transplantation using similar analysis in a population of kidney transplant recipients with a median graft survival of 1.4 years and follow-up time of 3.8 years [22]. In the modern era, when average death-censored graft survival following kidney transplantation exceeds 10 years [23], the finding that the survival benefit of re-transplantation persists despite a prolonged exposure to immunosuppression and CKD is of clear clinical relevance.

Re-transplantation is invariably challenging in this group as these individuals present both technical and immunological difficulties. Transplant recipients may exhibit a more rapid loss of kidney function in the months prior to ESRD than the transplant-naïve CKD population [24]. This study demonstrates that the median survival time after commencing dialysis is short, and presumably, the window of opportunity for transplantation is shorter again; this, combined with the rapidity of decline in graft function prior to transplant failure, necessitates a degree of urgency in the management of these recipients.

Over the past 40 years, 13% of kidney transplants performed at this centre have been re-transplants. This is comparable to the re-transplantation prevalence elsewhere [25]. In this study, 57 recipients with failed grafts were re-transplanted; these individuals were younger, had a reduced incidence of diabetes mellitus and cardiovascular disease, and were more likely to commence dialysis with permanent access than those who did not receive a second transplant. There were proportionally more living donor re-transplants than first transplants in this centre during the same period. Living donation offers high-quality kidneys and the opportunity for planned, pre-emptive re-transplantation [3]. Recipients with failing grafts are likely to have families who have witnessed the transformational impact of transplantation. These individuals may be motivated to donate a kidney, and this option should be pursued at an early stage.

Evidence is accumulating that management of CKD complications is unsatisfactory in transplant recipients with graft failure. In this study, targets for the management of anaemia and CKD mineral bone disease were not met. There was no evidence, however, that this influenced the likelihood of re-transplantation or survival. Additionally, the dialysis modality of choice for persons with a failed graft is unknown; 71% of patients in this cohort commenced haemodialysis. This is the largest study to compare survival outcomes between the dialysis modalities [26,27]. There was a trend towards improved survival in the peritoneal dialysis group, but this did not persist after multivariate analysis. This reflects other published work where an initial association between peritoneal dialysis and improved outcomes in transplant recipients has also been lost after adjustment for covariates [28,29].

### Strengths and limitations

Comprehensive clinical follow-up over 25 years is the first strength of this study. The low level of emigration from Northern Ireland creates an ideal environment for research into long-term transplant outcomes. Secondly, the wealth of prospectively collected data available allows detailed analysis of factors influencing both re-transplantation and survival and facilitates the investigation of how parameters at the time of transplant failure are associated with outcomes. Thirdly, the quantification of the effect of re-transplantation on recipient survival in a cohort with a median graft survival exceeding 10 years is novel and relevant. This is the largest study to date with detailed long-term follow-up of recipients with failed transplants.

The small number of recipients could be considered a limitation of this work. While this research lacks the power of a large, registry-based analysis, the detailed and comprehensive follow-up allows meaningful conclusions to be drawn. Secondly, as a result of the historic nature of this cohort, the mean age of the recipients is younger than in the present day. However, it is these individuals who are likely to experience graft failure and require a subsequent kidney transplant during their lifetime. In addition, the maintenance immunosuppressive regimen in the study period is not typical of the modern era. It is possible that recipients with graft failure who were transplanted in a later period may have higher rates of infection and cancer due to a greater cumulative immunosuppressive burden with the use of CNI and induction therapy. Thirdly, information on hypertension and blood pressure control was not consistently available for recipients during the follow-up period. This may have an impact on graft and recipient survival.

## Conclusion

The substantial progress in kidney transplantation over the past 30 years has enhanced both graft and recipient survival. However, the latter often exceeds the former, and it is increasingly common for transplant recipients to return to dialysis therapy. These individuals are young, have significant comorbidities, and their outcome on dialysis is poor.

There are many factors which influence survival after graft failure; some of these are predetermined, such as age, but others may be modified with strategies to minimise and manage complications in transplantation. The single factor which substantially improves survival and which is, at least partly, within the domain of the nephrologist to influence is re-transplantation. Improving outcomes for recipients with failed kidney transplants requires early recognition of inevitable graft failure, assessment and optimisation of comorbid conditions, investigation of the possibility of living kidney donation, and timely re-transplantation if feasible.

## Abbreviations

CKD: chronic kidney disease; RRT: renal replacement therapy; PTFE: polytetrafluoroethylene; AVF: arteriovenous fistula; EGFR: estimated glomerular filtration rate; CNI: calcineurin inhibitor.

## Competing interests

The authors declare that they have no competing interests.

## Authors’ contributions

JAM conceived the study, participated in the study design, collected the data, performed the statistical analysis, and drafted the manuscript. CCP participated in the study design and performed the statistical analysis. APM revised the manuscript. AEC collected the data and drafted and revised the manuscript. All authors read and approved the final manuscript.
